# Recycling of spent mushroom substrate: Utilization as feed material for the larvae of the yellow mealworm *Tenebrio molitor* (Coleoptera: Tenebrionidae)

**DOI:** 10.1371/journal.pone.0237259

**Published:** 2020-08-06

**Authors:** Tian-Hao Li, Peng-Fei Che, Chao-Ran Zhang, Bo Zhang, Asad Ali, Lian-Sheng Zang

**Affiliations:** 1 Jilin Engineering Research Center of Resource Insects Industrialization, Jilin Agricultural University, Changchun, China; 2 Engineering Research Center of Chinese Ministry of Education for Edible and Medicinal Fungi, Jilin Agricultural University, Changchun, China; 3 Department of Agriculture, Abdul Wali Khan University, Mardan, Pakistan; Chinese Academy of Agricultural Sciences Institute of Plant Protection, CHINA

## Abstract

Spent mushroom substrate is made from the waste remaining after the harvest of mushrooms. Here, we evaluated the potential of five spent edible fungi (*Auricularia cornea*, *Lentinus edodes*, *Pleurotus eryngii*, *P*. *citrinopileatus* and *P*. *ostreatus*) substrates as feed sources for *Tenebrio molitor* larvae. Young larvae did not survive on any substrate except the spent *L*. *edodes* substrate (36.7%). The survival rates in young larvae were similar among the different diets in which wheat bran or rice bran was replaced with 0, 20, 30, 40, 50, or 60% spent *L*. *edodes* substrate. The weights of the surviving larvae were decreased only when 70% of wheat bran and > 40% of rice bran was replaced with spent *L*. *edodes* substrate. In addition, the middle-aged larvae fed wheat bran only were significantly larger than those fed diets with 30~60% spent *L*. *edodes* substrate in dry feed, but the larvae of all treatments failed to pupate. Whereas the green feed was added in dry feed, there were no significant differences in pupal weight, pupation rate, pupal duration, adult emergence, or deformed adults among the three treatments in middle-aged larvae that were fed on diets containing 0, 30, or 40% spent *L*. *edodes* substrate. Collectively, these results suggest that spent *L*. *edodes* substrate has considerable potential to be used as a partial replacement (< 40%) of conventional feed for *T*. *molitor*, and spent mushroom substrate waste may be recycled as feed material for resource insects.

## Introduction

Mushrooms are a delicacy known for their nutritional value, and the history of mushroom use can be traced back to 4000 B.C. in Mycenaean Greece [1]. Since then, the use of mushrooms has mostly relied largely on the collection of naturally grown mushrooms. However, many countries can now produce and distribute large quantities of edible fungi produced through standardized cultivation techniques. For example, the total production of edible fungi reached 29 million tons in 2015 in China [2]. Since only edible parts of the fungi are harvested, the remaining substrate, better known as ‘spent mushroom substrate (SMS)’, contains edible fungal residues and approximately 75–85% of unused nutrients [3]. In general, for every 1 kg of edible fungi harvested, 3.25 kg of SMS is produced [4]. Thus, SMS is a class of nutritious organic waste that may be worth reusing.

The use of SMS has been studied from many different perspectives [5]. In terms of the scale of utilization, large amounts of SMS have been used as fertilizer, as SMS possesses all essential attributes of organic manure after recomposting by natural weathering or any other process [6, 7]. SMS has been used as a substrate for culturing the same or other edible fungi [8, 9] and has been used for producing bacteria [10]. Due to its loose porous structure and unique biological properties, SMS can also be used as a biosorbent [11, 12] in wastewater treatment [13] or for the microbial degradation of polycyclic aromatic hydrocarbons for environmental protection [14]. The other utilities of SMS include the prevention of biofouling [15], substrate for vermicomposting and pest management [5]. For example, the bacterium *Pseudomonas aeruginosa* from SMS was reported to effectively suppress the fungal disease gray leaf spot (blast) on perennial ryegrass turf [16].

Interestingly, SMS can be used as animal feed, as shown in feed research involving livestock [17, 18] and poultry [19]. Xu et al. [20] showed that an SMS level of 6.5% in the dry matter of cattle’s diet can be recommended for silage based on the total mixed ratio. Considering their feeding characteristics and food chain relationship, omnivorous or saprophytic insects can be more efficient at degrading organic wastes than large organisms [21, 22], with lower greenhouse gas emissions [23].

The yellow mealworm, *Tenebrio molitor* L. (Coleoptera: Tenebrionidae), is a resource insect worldwide. Larvae are widely used as food sources in animal rearing as well as in human diets due to their high protein content and nutritional value [24, 25]. For example, they are typically used as a food for insectivorous mammals, birds, reptiles, amphibians and fishes in captivity [25, 26]. Conventionally, mealworms are raised on wheat bran. In South Korea, Kim et al. [27] first reported the use of SMS for the rearing of *T*. *molitor* larvae (TML). They showed that in all the groups fed different proportions of *Pleurotus eryngii* SMS added to what bran, the survival rates were similar to that in the control group, which were fed with 100% wheat bran. However, larvae in the treated groups weighed significantly less than those in the control group [27]. In another experiment, however, TML were fed *Flammulina velutipes* SMS to replace wheat bran, and this species of mushroom was associated with better results than those in the former study. The larval development times were 11.36, and 11.71 weeks, the average pupal weights were 0.12, and 0.12 g, and the pupation rates reached 78.33, and 78.00% when TML was fed wheat bran with 40 and 50% SMS, respectively [27]. Additional studies are needed to provide useful information for the potential use of different edible fungi substrates, especially those with high local consumption, as feed for TML.

This study aimed to investigate the potential of SMS from several major edible fungi as feed for TML in China, where the edible mushroom industry has been experiencing rapid development since 1978. Industrial production technologies started to advance after the new millennium, with nearly 50 different species of edible fungi being commercialized in China [28]. Among them (in order of total yield), *Lentinus edodes*, *Pleurotus ostreatus* and *Auricularia auricula* are the top three edible mushrooms [28], and *Flammulina velutipes* and *Pleurotus eryngii* are the two most industrialized mushrooms [29]. Some edible fungi, such as *Pleurotus citrinopileatus*, are only locally produced, and Jilin Province is the second largest producing region of *P*. *citrinopileatus* [30]. Some new edible fungal species, such as *Auricularia cornea*, have also recently been developed and compete with *A*. *auricula* on the market [31]. We first investigated the feeding adaptability of TML to the SMSs of the five most important fungi (*A*. *cornea*, *L*. *edodes*, *P*. *citrinopileatus*, *P*. *eryngii* and *P*. *ostreatus*) in China. After we found that the *L*. *edodes* SMS might have potential to partially replace conventional feed, we determined an optimal proportion for the substitution. Because young larvae are highly susceptible to feed restriction, we evaluated the effects of the feed on the growth and development of young and middle-aged larvae separately.

## Materials and methods

### Insect colony

All insect rearing and experiments were carried out in an insectary under suitable conditions (26 ± 1 °C, 55 ± 3% R.H, 10L:14D using 450 ± 50 lux white LED light). TML and adults were initially obtained from Xinchong Aquaculture Co., Ltd, China in 2018. Groups of 800–900 adult beetles were kept in wooden boxes (500 × 320 × 200 mm) [32] for 45–50 days [33]. The inner surface of the box was smoothed using scotch tape to prevent the beetles from escaping. The bottom of the box was made of stainless-steel mesh with a mesh diameter of 2 mm. Paper was placed under the box to collect eggs. Eggs for colony maintenance were collected twice per week, and eggs for experiments were collected 24 hours prior to the experiments. Larvae were reared on an artificial diet (85% wheat bran, 10% Chinese cabbage and 5% cucumber). Newly hatched larvae were first maintained on egg papers in small plastic boxes (360 × 250 × 110 mm) for 15 days and then transferred to large plastic boxes (440 × 320 × 130 mm) after the papers were removed, where they were reared for another 30 days. Afterwards, the larvae were moved to the largest plastic boxes (570 × 400 × 100 mm) and reared until pupation [34]. Pupae were collected daily in plastic boxes (240 × 180 × 70 mm). Large and healthy adults were used for reproduction. The colony was maintained for multiple generations prior to the experiments.

### Preparation of SMS and conventional feed

The spent substrates of the five edible fungi (*A*. *cornea*, *L*. *edodes*, *P*. *citrinopileatus*, *P*. *eryngii* and *P*. *ostreatus*) were provided by the Engineering Research Center of the Chinese Ministry of Education for Edible and Medicinal Fungi in Changchun, China. The raw materials of these edible fungal culture media as well as their cultivation and harvesting conditions are shown in Table 1. After natural air drying, each substrate was crushed by a pulverizer (Laobenhang Multifunctional Crusher, Yongkang Boou Hardware Products Co., Ltd., China) at 35000 r/min for 20 seconds. The powder of each substrate was then separated into two parts using a sieve (1.25-mm diameter), and the finer portion was preserved for the experiments.

**Table 1:** Raw materials of edible fungal culture medium and cultivation and harvesting conditions.

Fungal species	Raw culture medium	Cultivation conditions			Harvest times
		Temp. (°C)	RH (%)	Duration (days)	
*Auricularia cornea*	88% saw dust, 10% wheat bran, 1% soybean powder, 0.5% lime powder and 0.5% gypsum	24 ± 5	90 ± 5	60	3
*Lentinus edodes*	78% saw dust, 16% wheat bran, 2% gypsum, 1.5% corn meal, 1.5% sugar, 0.5% urea and 0.5% calcium superphosphate	22 ± 5	90 ± 5	60	3
*Pleurotus citrinopileatus*	80% corncob powder, 18% wheat bran, 1% lime powder and 1% gypsum	22 ± 3	90 ± 5	60	3
*Pleurotus eryngii*	48% saw dust, 24% corncob powder, 20% wheat bran, 5% corn meal, 2% gypsum and 1% sugar	16 ± 3	90 ± 5	28	1
*Pleurotus ostreatus*	92% cottonseed hull, 5% wheat bran, 1% soybean meal, 1% sugar and 1% gypsum	20 ± 5	90 ± 5	60	3

Raw wheat bran or rice bran was purchased from a local market in Changchun, China. The raw materials of the wheat or rice bran were also separated into two parts using a sieve (1.25- mm diameter), and only the finer part was used for the experiments. For standard rearing, appropriate amounts of green feed, such as Chinese cabbage or watermelon peels, were added to the dry feed [35, 36]. We used fresh watermelon peels, which were also collected from a local market prior to each experiment, as green feed.

### Suitability of SMS as feed for young larvae

Each SMS of the five different fungi (Table 1) was tested for its suitability for the growth and development of young TML compared to conventional feed comprising wheat or rice bran. The testing methods were similar for each SMS group. For each test replicate, 50.0 ± 0.1 g of feed was weighed using an electronic balance (FB224, Shanghai Shunyu Hengping Scientific Instrument Co., Ltd., China. Max = 220g, Min = 0.01g, e = 1mg, d = 0.1mg) and then placed in a plastic box (140 × 105 × 50 mm) covered by a 50 × 50 mm mesh air vent lid. All boxes were placed in the insectary for 24 hours prior to the introduction of TML. This process was necessary to keep the powder temperature and humidity the same as that in the environment to avoid unnecessary stimulation of the larvae. Afterwards, 30 first instar TML with samilar size were introduced into each box and reared for 30 days to assess survival. The total body mass of the surviving larvae was measured. five replicates for each feed treatment were conducted.

### Effect of mixed dry feed on the growth and development of young larvae

**Fig 1:**
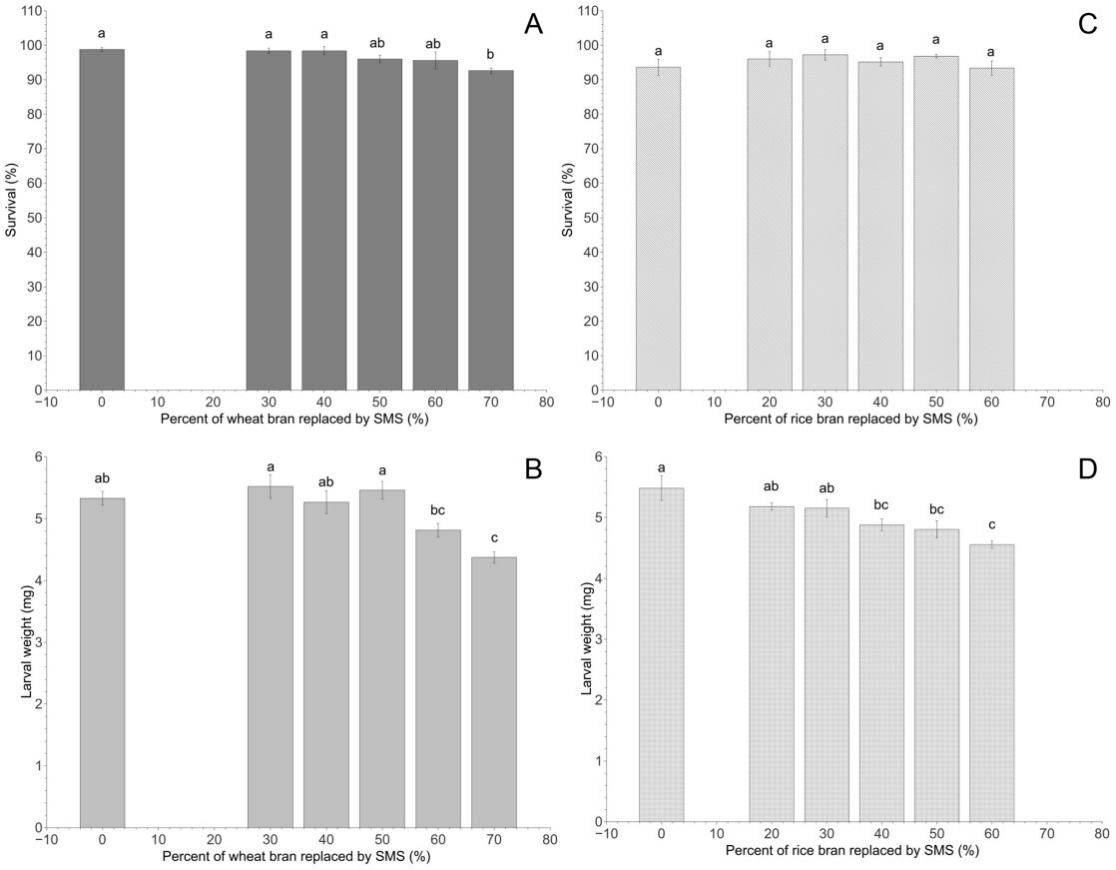
Biological parameters of young TML after a 30-day rearing period with different feed formulations: Survival (A) and larval weight (B) after rearing on different proportions of *L*. *edodes* SMS and wheat bran; survival (C) and larval weight (D) after rearing on different proportions of *L*. *edodes* SMS and rice bran. Means (± SE) are presented. Different lowercase letters above each bar in a given group indicate significant differences (*P* < 0.05, ANOVA, Tukey’s HSD test).

Based on the results of the above experiment, *L*. *edodes* SMS, which was the most suitable, was further evaluated for its potential as a partial substitute for conventional dry feed comprising wheat bran or rice bran. To determine the optimal proportion for the substitution, the experiment consisted of 12 different diet treatments, each weighing a total of 50.0 ± 0.1 g. The first six diets were formulated as dry wheat bran with 0 (control), 30, 40, 50, 60, and 70% *L*. *edodes* SMS, while the other six diets consisted of rice bran with 0 (control), 20, 30, 40, 50, and 60% *L*. *edodes* SMS (Fig 1). As described above, each feed was placed in a plastic box (140 × 105 × 50 mm) in the insectary for 24 hours prior to the introduction of 50 first instar TML with similar size. After a 30-day rearing period, the number of surviving larvae was counted, and the total body mass of the surviving larvae was measured. Five replicates for each of the 12 diet treatments were conducted.

### Effect of mixed feed on the growth and development of middle-aged larvae

To determine the effect of the mixed feed formulations on the growth and development of middle-aged TML, five diets consisting of wheat bran with 0 (control), 30, 40, 50 and 60% *L*. *edodes* SMS were examined. The number of *T*. *molitor* instars varied from 9 to more than 20, depending on rearing conditions [37], but most of them developed through 13 to 16 instars [38, 39]. In this experiment, we considered 9^th^ instars as middle-aged larvae, and larvae of this age were used for the tests. As described above, each test replicate consisted of 50.0 ± 0.1 g feed placed in a plastic box (140 × 105 × 50 mm). After 24 hours, 30 larvae (mean mass 19.4 ± 1.3 mg) were introduced into each box and reared for 45 days.

To consider the possible effect of green feed on insect growth and development, each treatment was further divided into two groups: group A was fed dry feed only, whereas group B was fed dry feed and 2.0 ± 0.1 g of fresh watermelon peels. The watermelon peels were provided every three days until the end of the 45-day rearing period (i.e., a total of 15 times). All larvae were monitored daily, and once the larvae had pupated, the pupal weight along with the pupation date were recorded. Pupae were transferred to tubes (2-cm diameter, 8-cm length) and reared individually until adult emergence. The emergence dates and whether the adults were normal or deformed (had any defect in appearance or mobility) were recorded. At the end of the 45-day rearing period, if the larvae had not yet pupated, the number of surviving larvae and the total body mass of the larvae were recorded. Five replicates for each feed treatment were conducted.

### Data analysis

The average larval or pupal weight of the TML in each replicate was obtained by dividing the total body mass by the number of surviving insects. For each measured parameter, data were analyzed using one-way ANOVA, and means were separated using Tukey’s HSD test at *P* < 0.05. All data were subjected to a normality test (Ryan-Joiner test) prior to ANOVA. All statistical analyses were performed using Minitab 19 (Minitab^®^ Statistical Software, State College, PA, USA). The figures were plotted using QtiPlot 0.9.8.9 svn 2288.

## Results

### Suitability of SMS as feed for young larvae

The survival rates of newly hatched TML were significantly different with different feed formulations (*F*_6,28_ = 163.0, *P* < 0.001) (Table 2). The percent survival of larvae fed wheat bran (96.7%) and rice bran (94.7%) was similar and was significantly higher than that of larvae fed diet supplemented with *L*. *edodes* SMS (36.7%). Only a small percentage (1.3%) of the larvae survived on the *P*. *eryngii* SMS, and no larvae survived on the other substrates.

**Table 2:** Percent survival and body mass of young *T*. *molitor* larvae after a 30-day rearing period on different singular diets.

Feed type	Survival (%)^1^	Larval weight (mg)^1^
Wheat bran	96.7 ± 1.1 a	6.84 ± 1.15 a
Rice bran	94.7 ± 1.7 a	3.56 ± 1.25 b
*Lentinus edodes* SMS	36.7 ± 9.1 b	0.53 ± 0.42 c
*Pleurotus eryngii* SMS	1.3 ± 0.8 c	—^2^
*Pleurotus citrinopileatus* SMS	0.0 ± 0.0 c	—^3^
*Pleurotus ostreatus* SMS	0.0 ± 0.0 c	—^3^
*Auricularia cornea* SMS	0.0 ± 0.0 c	—^3^

^1^Values (mean ± SE) followed by different letters within the same column are significantly different (*P* < 0.05, ANOVA, Tukey’s HSD test).

^2^ Larvae were too small to be weighed.

^3^ No larvae survived.

The feed formulation also affected the weight of surviving larvae (*F*_2,12_ = 1652.9, *P* < 0.001) (Table 2). The larvae fed on wheat bran had the greatest body mass (6.84 mg), which was significantly higher than those fed on rice bran (3.56 mg) or *L*. *edodes* SMS (0.53 mg). A few larvae that survived on the *P*. *eryngii* SMS were too small to be weighed.

### Effect of mixed dry feed on the growth of young larvae

Mixing wheat bran with different proportions (0~70%) of *L*. *edodes* SMS significantly affected the survival of first instar TML (*F*_5,24_ = 3.4, *P <* 0.05) and the weight of surviving larvae (*F*_5,24_ = 9.6, *P* < 0.001). However, mixing rice bran with different proportions (0~60%) of *L*. *edodes* SMS did not significantly affect the survival of first instar TML (*F*_5,24_ = 0.9, *P* = 0.520); it only affected the weight of surviving larvae (*F*_5,24_ = 6.5, *P* < 0.05) (Fig 1). Larvae fed wheat bran alone or mixed with 30~60% *L*. *edodes* SMS had a higher survival rate and gained more weight than those fed wheat bran mixed with 70% *L*. *edodes* SMS. Similarly, SMS should only replace a small proportion of rice bran; larvae fed on the rice bran mixed with 40~60% *L*. *edodes* SMS were smaller than those fed on the rice bran alone or rice bran mixed with 20 or 30% *L*. *edodes* SMS (Fig 1).

### Effect of feed formulations on the growth and development of middle-aged larvae

**Fig 2:**
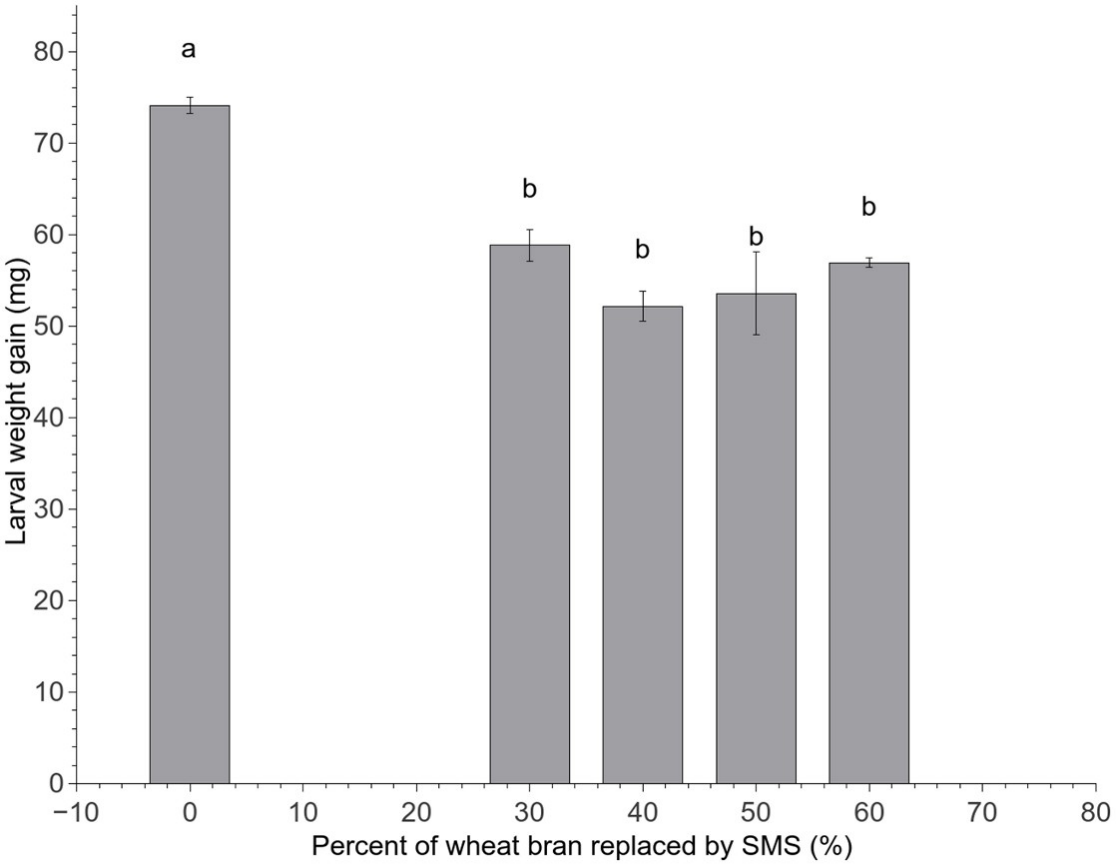
Larval weight gain of middle-aged TML after a 45-day rearing period with different dry feed formulations (Group A). Means (± SE) are presented. Different lowercase letters above each bar in a given group indicate significant differences (*P* < 0.05, ANOVA, Tukey’s HSD test). No larvae had died or pupated at the end of the test.

None of the middle-aged TML in all treatments died and pupated after a 45-day rearing period on dry feed only. The larvae fed wheat bran only were significantly larger than those fed wheat bran mixed with 30~60% *L*. *edodes* SMS (*F*_4,20_ = 14.65, *P* < 0.001) (Fig 2).

**Fig 3:**
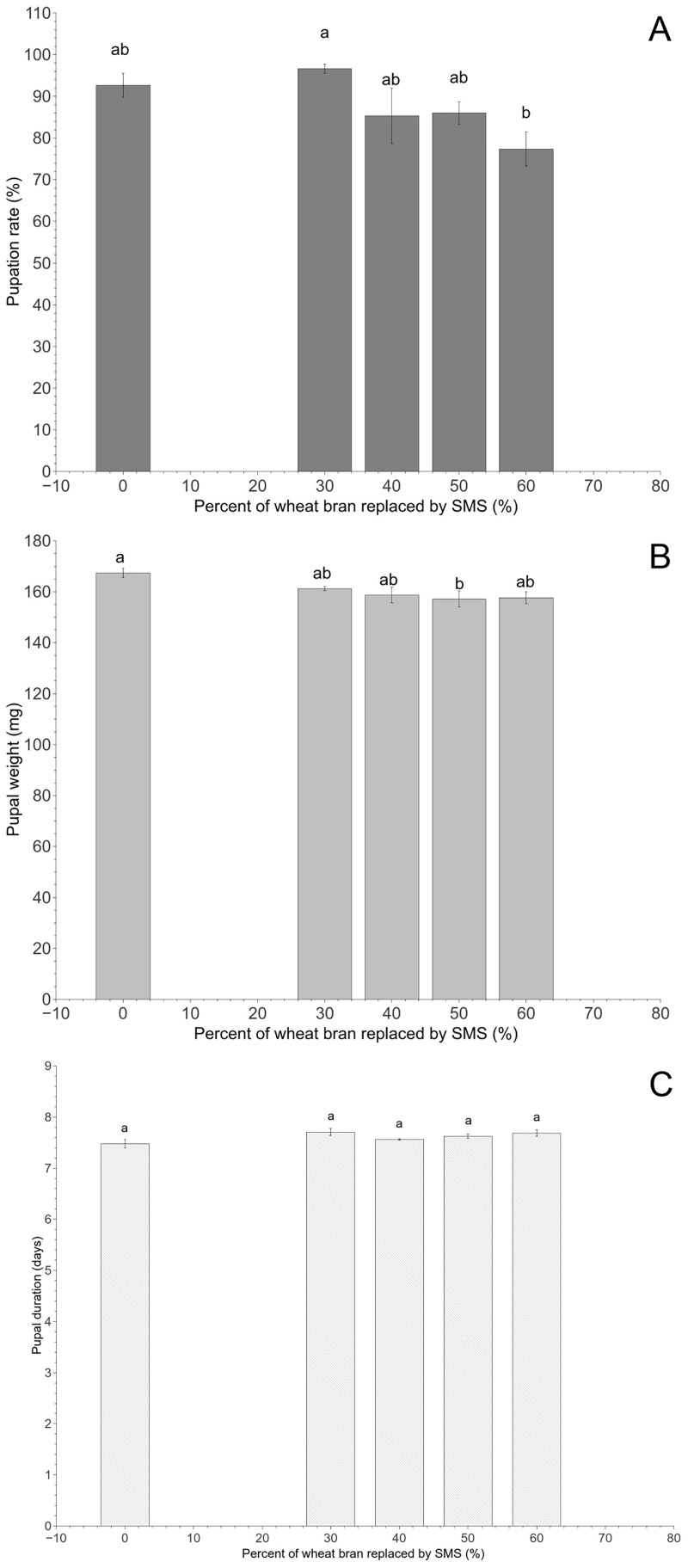
Pupation rate (A), pupal weight (B) and pupal duration (C) in middle-aged *T*. *molitor* larvae after 45 days of rearing on different dry feed formulations and watermelon peels (Group B). Means (± SE) are presented. Different lowercase letters above each bar in a given group indicate significant differences (*P* < 0.05, ANOVA, Tukey’s HSD test).

When watermelon peels were added to the dry feed, however, most of the larvae developed into pupae within 45 days. Significant differences among different diet treatments were detected in the pupation rate (*F*_4,20_ = 3.56, *P* < 0.05) and pupal weight (*F*_4,20_ = 3.08, *P* < 0.05) (Fig 3) but not in the pupal duration (*F*_4,20_ = 2.48, *P* = 0.077) (Fig 3), adult emergence rate (*F*_4,20_ = 1.38, *P* = 0.278) or percentage of deformed adults (*F*_4,20_ = 1.76, *P* = 0.176) (Table 3). In general, the pupation rate was lower and the pupal size was smaller in larvae fed wheat bran mixed with 60% *L*. *edodes* SMS than in those fed wheat bran alone or wheat bran mixed with 30~50% *L*. *edodes* SMS (Fig 3).

**Table 3:** Adult yellow mealworms obtained from different diets (dry feed plus watermelon peels).

Dry feed composition (%)		Adult emergence (%)^1^	Deformed adults (%)^1^
*L*. *edodes* SMS	Wheat bran		
0	100	97.8 ± 1.4 a	2.26 ± 0.93 a
30	70	93.8 ± 1.3 a	3.00 ± 1.43 a
40	60	93.7 ± 3.2 a	3.25 ± 0.87 a
50	50	97.6 ± 1.0 a	0.00 ± 0.00 a
60	40	97.3 ± 1.1 a	2.55 ± 1.05 a

^1^Values (mean ± SE) followed by different letters within the same column are significantly different (*P* < 0.05, ANOVA, Tukey’s HSD test).

## Discussion

This study showed the potential of *L*. *edodes* SMS to be used in feed for TML rearing; the other four tested substrates appeared to be unsuitable as feed for TML. Although young TML were able to survive on *L*. *edodes* SMS alone, the nutrition provided by the *L*. *edodes* SMS alone appeared not to meet the long-term development needs of the larvae. The survival rate was significantly reduced, and the surviving larvae gained substantially less body weight than those fed pure and conventional feeds (wheat bran or rice bran). However, mixing the *L*. *edodes* SMS with conventional feed significantly increased the survival and weight of young TML. In our study, we found that the survival rate of young TML fed on *P*. *eryngii* SMS alone was extremely low (1.3%). In an early study, Kim et al. [27] showed the potential of *P*. *eryngii* SMS as a substitute feed for TML, although the performance of the larvae on the *P*. *eryngii* SMS was reduced when compared to those fed on the control treatment. Thus, the *L*. *edodes* SMS could be used as only a partial substitute for conventional feed. It is not clear why TML failed to survive on the substrates of the other four edible fungi. Obviously, the spent substrate from different mushrooms vary in their physical, chemical and biological properties, and each one could have its own specific utility. Additional detailed studies are needed to identify some key nutritional components of SMS that could affect the survival and development of TML.

Middle-aged TML performed almost equally well when fed diets with 30 or 40% *L*. *edodes* SMS as those fed the pure and conventional diets in terms of various fitness parameters. It is thus appropriate to substitute 30 ~ 40% the conventional diet with *L*. *edodes* SMS. We also found that no addition of green feed to dry feed delayed the pupation of TML. Our conclusion is generally consistent with that of a previous study [27], which showed that the spent substrates of *F*. *velutipes* and *P*. *eryngii* [40] were suitable as feed for TML. Their study also suggests that 40~ 50% replacement of wheat bran by these substrates is appropriate for the rearing of TML. SMS of *P*. *eryngii* and *L*. *edodes* were also successfully used as food sources for larvae of the white-spotted flower chafer *Protaetia brevitarsis seulensis* (Coleoptera: Cetoniidae), a resource insect known to have important medicinal properties, such as anticancer activity [41]. Cai et al. [42] also reported the use of *F*. *velutipes* SMS as feed for the black soldier fly *Hermetia illucens* (Diptera: Stratiomyidae). Although detailed information on the suitability of SMS for *H*. *illucens* was not available in this brief study, it provides another example of the potential use of SMS for the rearing of different insects.

We also demonstrated that rice bran as well as wheat bran was suitable as dry feed or as a dry feed mixture with *L*. *edodes* SMS for TML. In rice-producing regions such as northeastern China, rice bran is not only cheaper but also easier to obtain than wheat barn and requires lower transportation costs than wheat bran. Therefore, rice bran could emerge as a valuable feed for TML. The only disadvantage is that the rice bran is not tolerant to long-term storage and, if deteriorated, produces aldehydes and ketones that can damage the livers of animals [43]. For this reason, a large amount (80%) of rice bran is not used effectively or is discarded as waste in China [44].

The use of SMS could greatly reduce the costs of rearing TML, as the material is cheaper than conventional feed and is easy to obtain from the mushroom industry. Although TML are mainly used as poultry or fish feed in most countries, they are also directly consumed by humans [25]. One concern might be the safety of the direct consumption of TML or the consumption to other animals that fed on TML reared from SMS. However, SMS is a residue from human food production, and it is produced in the same way as the edible part of the mushroom. If it is processed promptly and stored properly, there should be minimal food safety risks. Furthermore, SMS is provided as a dry feed to TML, and under dry conditions, the feed is less likely to deteriorate. The demand for animal protein is expected to rise by 70–80% between 2012 and 2050, and the current animal production sector is already causing major environmental degradation [45]. Edible insects, such as TML, are a highly sustainable source of animal protein [25]. In addition, TML can also be used for the rearing of some beneficial insects for biological control. For example, *T*. *molitor* pupae have been used as an alternative host for *Chouioia cunea* [46], an important natural predator that is mass-reared and released to control the invasive fall webworm (*Hyphantria cunea*) in China. It is also an alternative rearing host for *Scleroderma guani* [47, 48], which is a major parasitoid used for the control of the Asian longhorned beetle *Anoplophora glabripennis* in China [49].

Insects play an important role in the decomposition and transformation of organic wastes in nature. There is increasing concern that the accumulation of large quantities of organic agricultural or horticultural waste, such SMS, could have harmful impacts on the environment if the wastes are not recycled promptly and properly [50]. Large portions of these wastes have been traditionally reused to produce edible fungi and fertilizers. These wastes could also be used in the form of feed to produce insect biomass while to composting raw materials, hence contributing to a clean and friendly environment. For example, *H*. *illucens* has been used for recycling restaurant waste, vegetable waste [51], livestock and poultry manure [52]. It should be noted, however, that *H*. *illucens* prefers food with a high moisture content. Under wet conditions, SMS-containing feed could deteriorate rapidly, creating environmental issues such as a bad odor. In contrast with *H*. *illucens*, TML prefer dry feed, so there will be no problems with feed decay. Our results suggest that some spent mushroom substrates, such as *L*. *edodes* SMS, can potentially be used as feed material for TML and other resource insects.

## Supporting information

S1 Raw data (XLSX)
